# Development of a growth factor bioavailability enhanced allograft (GFBA) for bone regeneration

**DOI:** 10.1007/s10561-025-10206-y

**Published:** 2026-01-22

**Authors:** Marie-Soleil R. Smith, Sowmya Shivanna, Yakup Kohen, Shiva Naseri, Yasmin Mawani, Sean A. F. Peel

**Affiliations:** 1 Red Rock Regeneration Inc, Toronto, ON Canada; 2 Department of Medical Biophysics, Temerty Faculty of Medicine, University of Toronto, Toronto, ON Canada; 3 Princess Margaret Cancer Centre, University Health Network, Toronto, ON Canada; 4 Faculty of Dentistry, University of Toronto, Toronto, ON Canada

**Keywords:** Demineralized Bone Matrix, Natural Matrix Protein, Osteoinduction, Bone Regeneration, Orthobiologics, Bone graft substitutes

## Abstract

Bone morphogenetic protein (BMP) stimulated osteoinduction is critical for bone regeneration. Human demineralized bone matrix (DBM) has been one of the most widely used bone graft substitutes, but its osteoinductive potential is weak and clinical effectiveness limited in part due to ineffective processing methods. Here, we describe a novel process designed to enhance allograft bioactivity by increasing the bioavailability of the native BMPs present within the matrix the product of which we call Natural Matrix Protein® (NMP®). BMP-7 release was quantified from NMP and DBM prepared from both bovine and human cortical bone. In both species, NMP significantly increased BMP-7 levels in acidic and physiologic extracts compared to DBM. NMP also demonstrated sustained release of BMP-7 and total protein for up to 12 weeks in simulated body fluid. Osteoinductive potential was evaluated *in vitro* using C2C12 cells and osteoinductivity *in vivo* in the athymic rat model. Direct treatment of cells with NMP *in vitro* produced a greater than tenfold increase in alkaline phosphatase activity at 40 mg/mL. *In vivo*, human NMP showed increased osteoinductivity compared to human DBM histologically, and the recovered NMP explants had significantly more mineralized bone and a higher bone volume fraction compared to DBM and to Infuse® Bone Graft (105 µg rhBMP-2 on an absorbable collagen sponge) as measured by microCT. These findings demonstrate that the novel NMP process reproducibly increases BMP-7 bioavailability, that NMP implants produce sustained BMP and protein release and have marked increase in osteoinductive activity.

**Supplementary Information:**

The online version contains supplementary material available at 10.1007/s10561-025-10206-y.

## Introduction

Autologous bone grafts are considered the gold standard for filling bone defects and promoting bone repair. However, their use is limited by availability, variable resorption, and associated donor site morbidity, leading to a widespread use of bone graft substitutes. Allogenic bone is the second most commonly donated tissue worldwide and was used in over 2 million bone grafting procedures globally in 2022 (GlobalData 2023). Allogenic bone grafts are used clinically to fill defects and promote fusion, serving as an osteoconductive scaffold and, when demineralized, potentially providing limited osteoinductive activity depending upon the demineralization and other processing procedures utilized (Wolfinbarger et al. 2008).

The osteoinductivity of demineralized bone matrix (DBM) is primarily attributed to the presence of bone morphogenetic proteins (BMPs), where higher BMP content has been correlated with greater bone-forming activity (Bae et al. 2010; Gruskin et al. 2012; Murray et al. 2007). Less than 5% of BMPs can be extracted from mineralized bone matrix and it is only after demineralization that BMPs become bioavailable and that the allograft bone may become osteoinductive (Pietrzak et al. 2011; Sampath & Reddi 1984). Additional growth factors in the matrix, including transforming growth factor-*β*1 (TGF-*β*1), fibroblast growth factor 1 (FGF-1), platelet-derived growth factors (PDGF), and vascular growth factor (VEGF), are present in DBM (Murray et al. 2007; Wildemann et al. 2007) and may aid in promoting bone-forming activity through stimulating cellular recruitment, angiogenesis, and matrix remodelling (Devescovi et al. 2008). However, the osteoinductive potential of DBM is generally considered to be weak and unreliable due to the low amounts of BMPs present within the matrix (Murray et al. 2007; Pietrzak et al. 2006) and differences in processing methods (Clokie et al. 2000).

When delivered *in vivo*, extracted and purified native BMPs do not induce bone formation unless combined with a carrier due to their high solubility. Even when adsorbed onto a collagen sponge, supratherapeutic doses are required to achieve bone formation compared to the physiologic amounts present in DBM (Peel et al. 2017; Sampath et al. 1982). Within the collagenous matrix of DBM, BMPs have been hypothesized to exist in two distinct compartments, either “loose” or “tight”, in which only BMPs residing in the loose compartment can be readily eluted or extracted (Han et al. 2005; Pietrzak et al. 2012). Further, Pietrzak reported that only ~ 20% of the extractable BMP-7 present in human DBM eluted into a physiologic buffer over prolonged (168 h) incubation (Pietrzak & Ali 2017), further reducing the amount of BMP available to act and thus limiting the osteoinductive activity of the DBM.

Urist and colleagues described the preparation of a bone extract containing a mixture of native BMPs and non collagenous proteins (BMP/NCP), which is poorly soluble at neutral pH, is highly osteoinductive and promotes bone repair *in vivo* (Behnam et al. 2005; Urist et al. 1987, 1983). The active BMP component dissociates from the insoluble components of the BMP/NCP complex at acidic pH (Peel et al. 2003). Despite its high osteoinductivity, BMP/NCP preparation requires large amounts of bone, the use of hazardous chemicals and low-temperature processing in the presence of protease inhibitors, each of which limit the ability of tissue banks to produce BMP/NCP according to the Urist process. Further, BMP/NCP must be combined with an osteoconductive scaffold, which provides both space filling and structural support when used as a bone void filler. This paper investigates the potential of increasing the bioavailable BMP content and enhancing the osteoinductive potential of bone allografts by forming a BMP/NCP complex associated with the insoluble collagenous bone matrix which is scaled for processing single donors at room temperature in the absence of toxic chemicals or protease inhibitors. We have named this novel process the NMP process and its product Natural Matrix Protein® (NMP®).

## Methods

### Preparation of DBM and NMP

All bone was debrided of soft tissue, defatted, and ground into particulates. Cortical bone particles (0.25 – 1.0 mm) of human or bovine origin were treated with 0.5 or 1.2M HCl for up to 1 h, followed by water and buffer rinses to remove the HCl. The resulting matrix was loaded into dialysis tubing and incubated with a proprietary (P/S) solution overnight, followed by water rinses to remove the proprietary solution. All steps were performed at ambient temperature and did not use protease inhibitors. Following this, test articles were lyophilized. All human test articles and any bovine test articles used in *in vitro* and *in vivo* osteoinductivity testing were sterilized via low dose gamma irradiation. Bovine femurs were sourced from Animal Technologies, Tyler, TX, United States. All human tissue was obtained from AATB registered tissue banks with documented consent for research use.

### Protein extraction

Extraction methods were performed according to a modified version of the method used by Pietrzak et al. (2012). Specifically, 100 mg of test article was incubated in 4 mL of extract solution at room temperature with mechanical agitation. After 22 ± 2 h the supernatant was separated from the matrix by centrifugation. Acid-soluble proteins, including BMPs associated with non-collagenous proteins (BMP/NCP) were extracted with 50 mM acetic acid (AcOH). Physiologic pH soluble BMPs and other proteins were extracted using either Tris Buffered Saline (pH 7.2; TBS) or Phosphate Buffered Saline containing 0.05% Tween-20 (pH 7.2; PBST). Test articles were extracted with 4 M guanidine hydrochloride in 50 mM Tris (pH 7.2; Gdn) or 4 M guanidine hydrochloride in 50 mM Tris containing 0.05% Tween-20 (pH 7.2; GdnT), to estimate the total extractable BMP-7. AcOH, Gdn and GdnT extracts were buffer-exchanged (Amicon Ultra-4, 10 kDa MWCO, Sigma-Aldrich, St. Louis, MO, United States) into PBS or ELISA kit diluent.

For extended release studies, 100 mg of human NMP was incubated in 4 mL simulated body fluid (Biochemazone, Leduc, AB, Canada) containing 0.05% Tween and 0.05% NaN_3_ (SBF) with mechanical agitation at 37 °C. At each time point the supernatants were separated from the matrix by centrifugation. Supernatant collections and buffer replacement were performed at each time point (Day 1, 3, 7, 14, 28, 56, and 89) for a total of 12 weeks. Following extraction, all samples were centrifuged, supernatants collected, and stored at –30 °C.

### Protein quantification

BMP-7 concentrations in thawed extracts were quantified via enzyme-linked immunosorbent assay (ELISA) using the Quantikine BMP-7 kit (R&D Systems, Minneapolis, MN, United States) according to the manufacturer’s instructions. Resulting concentrations (pg/mL) were converted to ng BMP-7 per gram of test article, accounting for extraction volume and initial test article mass.

Total protein concentrations in thawed extracts were measured using the Micro BCA™ Protein Assay Kit (Thermo Scientific, Waltham, MA, United States) according to the manufacturer’s instructions. Resulting concentrations (μg/mL) were converted to mg protein per gram of test article, accounting for extraction volume and initial test article mass.

### *In vitro* activity

BMPs redirect the differentiation of C2C12 mouse myoblasts from a myogenic to an osteogenic lineage (Katagiri et al. 1994). The osteoinductive potential of the test articles was assessed in the C2C12 assay by measuring the induction of alkaline phosphatase activity as previously described by Peel et al. (2003). Briefly C2C12 cells (ATCC, Manassas, VA, United States) were cultured in DMEM (Sigma-Aldrich, St. Louis, MO, United States) supplemented with 10% FBS (Life Technologies, Carlsbad, CA, United States). On day 0, cells were plated in 24-well plates at 12.5 × 10^3^ cells/well. On day 1, fresh media and either 20 or 40 mg of each lyophilized NMP test article (n = 4) were added directly to the wells. Controls included untreated cells, 20 mg or 40 mg of lyophilized inactive DBM (extracted with Gdn), and 50 ng/mL rhBMP-2 as an internal positive assay control. Cultures were maintained at 37°C in 5% CO_2_, with media replaced every 2–4 days. After 14 days, cells were rinsed with 1X TBS, lysed with MPER (Sigma-Aldrich), and centrifuged. The supernatant was collected and stored at –20 °C until assayed for alkaline phosphatase activity using para-nitrophenol phosphate.

### Animal models

All *in vivo* studies were performed in male athymic rats by IBEX Preclinical Research (Logan, UT, United States) in accordance with ASTM F2529-13 (ASTM Committee F04, 2013). In brief, human NMP and DBM samples were hydrated with sterile saline and loaded into 1 cc syringes. An XXS Infuse® Bone Graft kit (rhBMP-2 on an absorbable collagen sponge (ACS)) was prepared according to the manufacturer’s instructions (Medtronic, MN. Specifically, lyophilized rhBMP-2 was reconstituted with sterile water and 0.7mL of the resulting 1.5 mg/mL rhBMP-2 solution (the dose used clinically) was applied to the ½" × 2" (~ 2cc) collagen sponge for a minimum of 15 min. The sponge was then cut into 10 pieces, each containing approximately 0.105 mg rhBMP-2. Hind leg muscle pouches were implanted with 0.2 cc of rehydrated DBM (n = 11), NMP (n = 19), or Infuse (n = 10). Implants were recovered after 28 days, fixed in 10% formalin, scanned by microCT (Quantum FX, PerkinElmer, MA), demineralized, embedded in paraffin, sectioned along the long axis at the center of the recovered implant, and stained with hematoxylin and eosin (H&E).

### Histology

Histologically based osteoinductivity (OI) scores were determined following the ASTM F2529-13 protocol (ASTM Committee F04, 2013). In brief, slides were digitally scanned and.czi image files were analyzed using ZEISS ZEN 3.11 imaging software. Three sections were assessed for each explant. The largest visible cross-section was selected and a 600 × 600 µm grid overlaid each section. A blinded reviewer assessed each square, scoring 0 or 1. Squares with a score of 1 contained bone-forming elements, including new bone, osteoblasts, osteocytes, bone marrow, or cartilage. Percent new bone (% NB) was calculated as the number of positive squares divided by the total number of squares scored. Final OI scores were: 0 = no implant detected; 1 =  < 10% NB; 2 = 10–20% NB; 3 = 21–30% NB; 4 =  > 30% NB. Approximately 400 squares per section were counted.

### microCT

MicroCT analysis was performed by a blinded reviewer using Analyze 15.0 software. Images were reconstructed with voxel size of 0.05 mm. Scans were cropped and segmented into two objects: “explant” (voxels above background density) and “bone” (voxels > 100 mg/cc, as determined using a phantom). Mean intensity and volume were measured for each object individually and in combination. Percent new bone (% NB), a measure of the quality of new bone, was calculated by dividing bone volume by total volume (bone + explant) and multiplying by 100.

### Statistical methods

All statistical analyses were performed using MedCalc, GraphPad, or R. Data are reported as mean ± SD or median with interquartile range (IQR). Normality was assessed using the Shapiro–Wilk test. Group comparisons were made using one-way ANOVA with Bonferroni-Dunn post hoc correction or the Kruskal–Wallis test with Dunn’s correction. Pairwise comparisons were assessed by Student’s t-test or Mann–Whitney U test. All tests were 2-tailed with significance set to p > 0.05.

## Results

### NMP process forms BMP/NCP and enhances BMP bioavailability

**Fig. 1:**
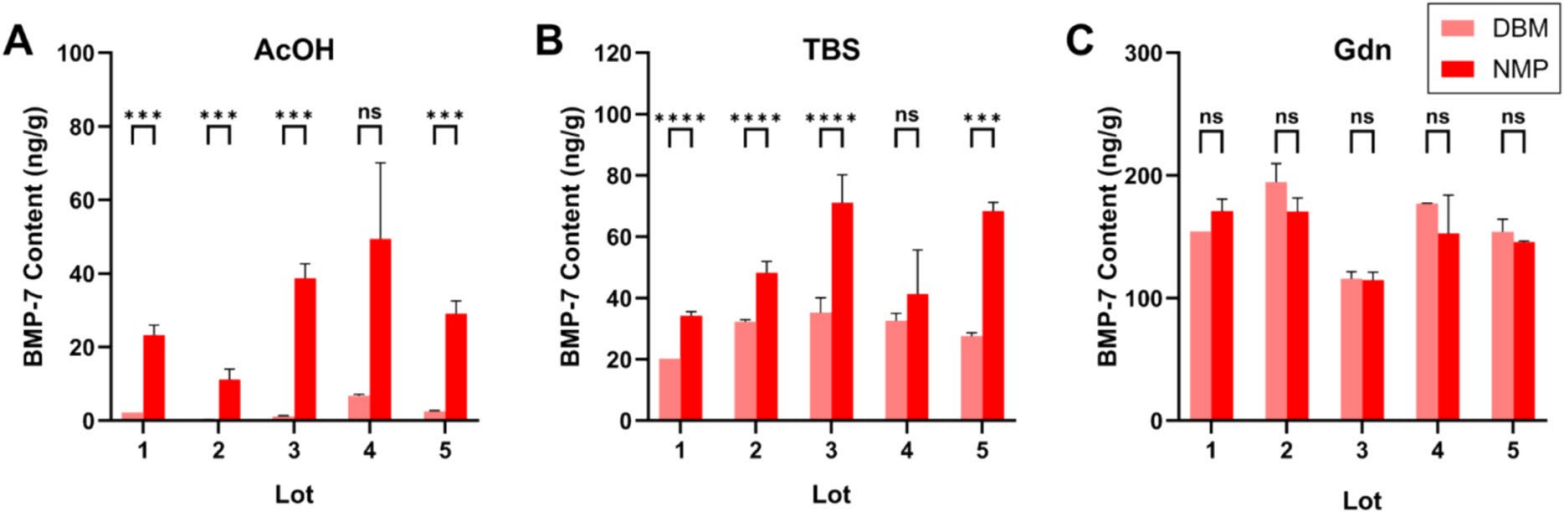
BMP-7 content in AcOH, TBS and Gdn extracts of NMP and DBM**.** BMP-7 content was quantified by ELISA from five independent lots of NMP or DBM processed bovine cortical bone particles in AcOH (**A**), TBS (**B**), or Gdn (**C**) extracts (N = 3–10, except Lot 1 DBM, N = 1). Data are shown as mean ± SD. Comparisons between DBM and NMP were made using Student’s t-test. *p < 0.05, **p < 0.01, ***p < 0.001, ****p < 0.0001, ns: non-significant

To first determine whether the NMP process produces a BMP/NCP complex and whether NMP has enhanced BMP-7 bioavailability, we examined the release of BMP-7 from bovine bone processed into NMP compared to bovine bone processed into DBM in three different extract solutions. NMP exhibited significantly higher BMP-7 content compared to DBM in both AcOH and TBS extracts (Fig. 1). On average, BMP-7 levels were increased > tenfold in AcOH extracts (*p* = 0.008; Fig. 1A) and ~ twofold in TBS extracts (*p* = 0.023; Fig. 1B). In contrast, BMP-7 levels in Gdn extracts remained comparable between NMP and DBM (*p* = 0.347; Fig. 1C). These differences were reproducible across five independent lots of bone processed at different times and by different operators, demonstrating consistency of the NMP process, although for 1 run the differences were not significantly due to the high variability in the BMP-7 ELISA results.

**Fig. 2:**
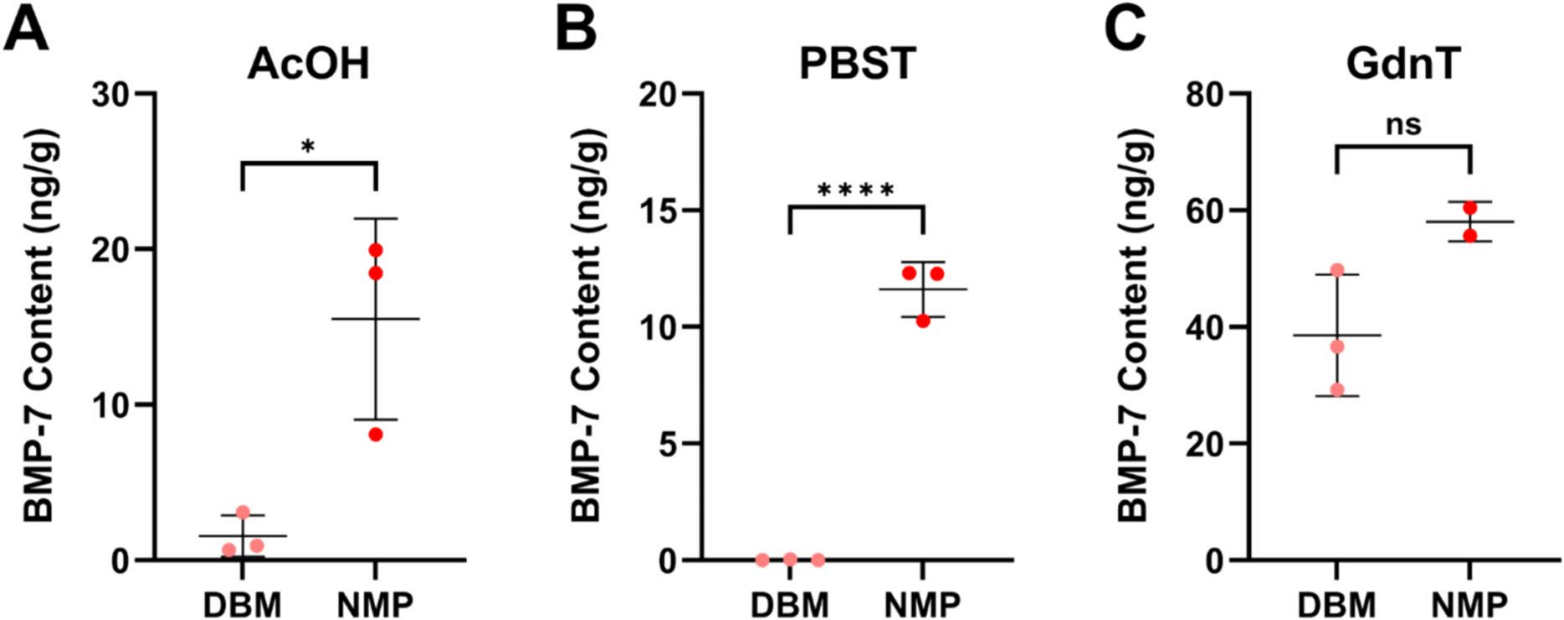
BMP-7 content in AcOH and TBS extracts of human NMP and DBM. BMP-7 levels were measured by ELISA in cortical bone particles from three independent donors processed as DBM or NMP in AcOH (A), TBS (B), and Gdn (C) extracts. Each data point represents an independent replicate; data are mean ± SD. For NMP Gdn, N = 2 due to sample loss during processing; all other groups, N = 3. Comparisons between DBM and NMP were made using paired Student’s t-test. *p < 0.05, **p < 0.01, ***p < 0.001, ****p < 0.0001, ns: non-significant

To determine whether the NMP process similarly produces BMP/NCP and enhances bioavailability in human bone, we compared BMP-7 release from NMP and DBM produced from human bone obtained from three different donors. Across all donors, NMP consistently demonstrated elevated BMP-7 levels in both AcOH (*p* = 0.022; Fig. 2A) and PBST extracts (*p* < 0.001; Fig. 2B) compared to DBM. BMP-7 content in GdnT extracts was not significantly different between NMP and DBM (*p* = 0.092; Fig. 2C). These findings demonstrate that the effect of the NMP process extends to human-derived bone allografts.

Although extractions reported here were performed in TBS for bovine samples and PBST for human samples, a separate study comparing BMP-7 levels in NMP extracted with both buffers showed the results were correlated (*r* > 0.539, *p* < 0.114; SI Fig. 1). Similarly, while the results here show bovine samples extracted in Gdn and human samples extracted in GdnT, a separate study comparing BMP-7 levels in NMP extracted with both Gdn and GdnT show strong correlation which neared significance (*r* = 0.937, *p* = 0.063; SI Fig. 1). Consequently, even though different buffers were used to evaluate bioavailability and total extractable BMP-7 in bovine and human test articles, the pattern of increased bioavailability without increase in total extractable BMP between NMP and DBM are consistent across bovine and human.

**Fig. 3:**
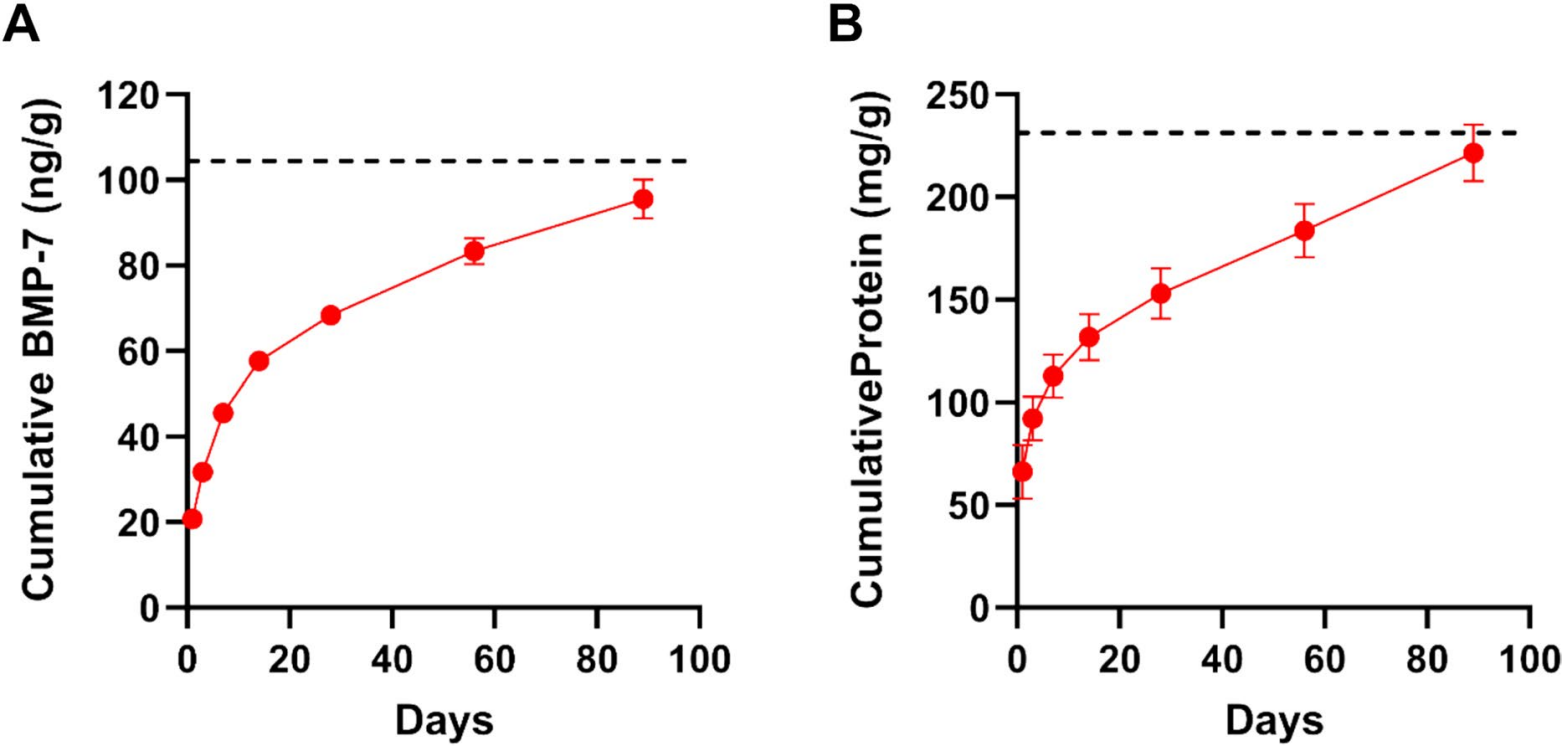
Cumulative release of BMP-7 and total protein from NMP *in vitro***.** Human NMP particles were incubated in simulated body fluid (SBF) at 37°C for 89 days. Cumulative BMP-7 (**A**) and total protein (**B**) release were quantified (n = 4). Data are shown as mean ± SD. Dashed line represents total BMP-7 as assessed via terminal GdnT extraction

To evaluate whether NMP processing of human bone results in long-term release of BMPs, NMP (n = 4) was incubated in SBF at 37 °C for 89 days. BMP-7 was released rapidly during the initial 24 h period, with an average release rate of 0.863 ng/g/hour (Fig. 3A). Between 56 and 89 days, BMP-7 release persisted at a reduced but measurable rate of 0.015 ng/g/hour, indicating sustained elution from the matrix. Total protein release followed a similar pattern, with an initial rate of 2.769 mg/g/hour and a final rate of 0.044 mg/g/hour over the final 32 days (Fig. 3B). Together, these data suggest that NMP processing generates both an early burst release of BMPs followed by a prolonged release phase.

### NMP demonstrates osteoinductive potential in the C2C12 *in vitro* assay

**Fig. 4:**
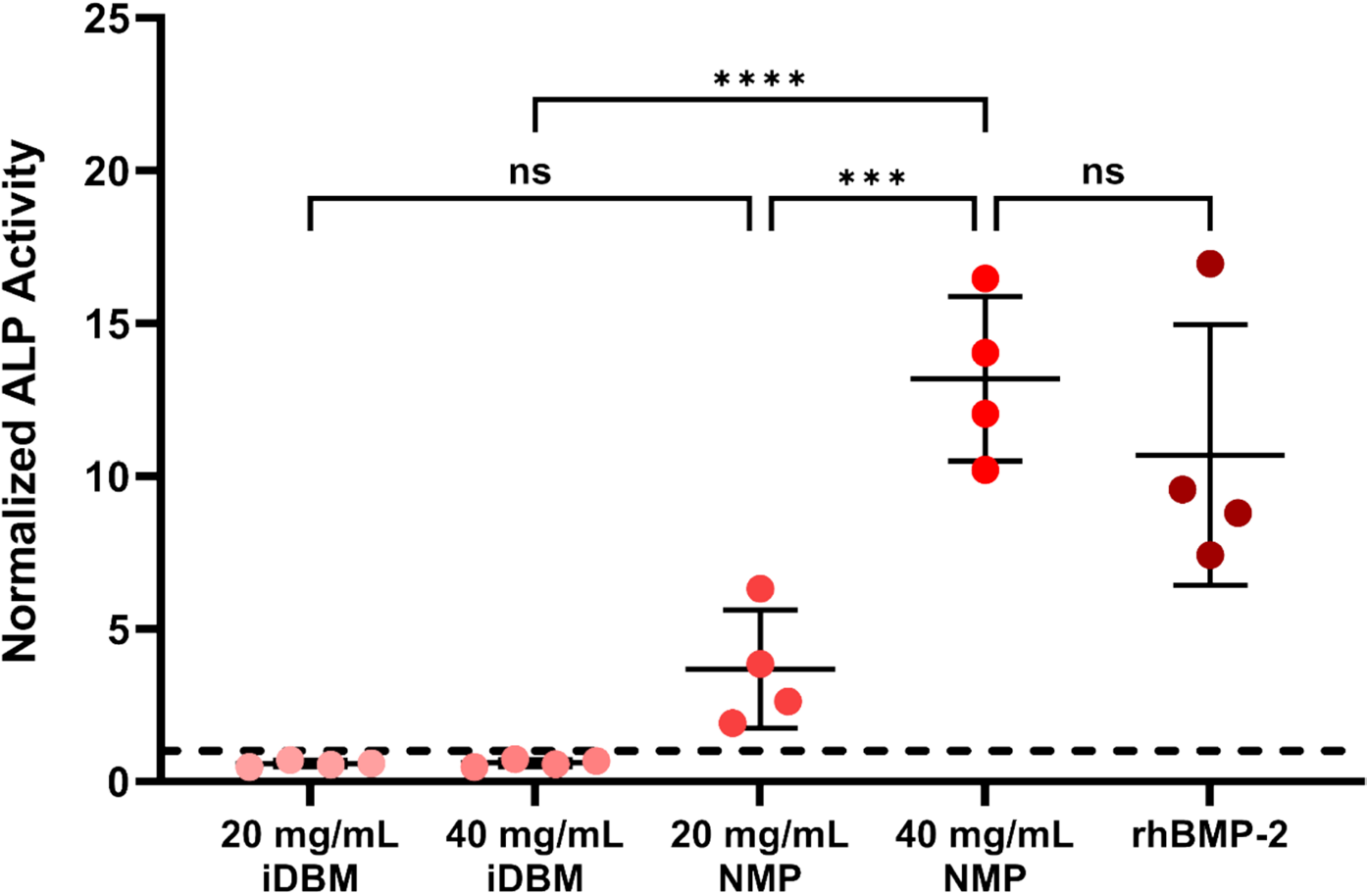
NMP is osteoinductive *in vitro*. C2C12 cells were cultured for 14 days with iDBM (20 or 40 mg/mL), NMP (20 or 40 mg/mL), or rhBMP-2 (50 ng/mL). Osteoinductivity was assessed by alkaline phosphatase (ALP) activity. Each symbol represents an independent replicate normalized to the cells-only control (dashed line). Data are mean ± SD. Comparisons were made by one-way ANOVA with Bonferroni correction. *p < 0.05, **p < 0.01, ***p < 0.001, ****p < 0.0001, ns: non-significant

To examine whether the NMP produced has osteoinductive potential, we conducted direct incubation of inactive DBM (iDBM; 20 and 40 mg/mL), human NMP (20 and 40 mg/mL), and rhBMP-2 (50 ng/mL) on C2C12 cells and assessed osteoinductive potential by measuring the increase in ALP. iDBM at both 20 and 40 mg/mL did not increase ALP activity compared to cells-only controls (Fig. 4). In contrast, NMP treatment produced a clear dose-dependent effect, with 20 mg/mL NMP yielding an approximately fourfold (non-significant) increase and 40 mg/mL NMP resulting in a > tenfold increase in ALP activity (Fig. 6; *p* < 0.001). At 40 mg/mL, NMP achieved ALP activity levels comparable to those observed with 50 ng/mL rhBMP-2. These findings demonstrate that, unlike iDBM, NMP exhibits a dose dependent osteoinductive potential, reaching levels on par with recombinant BMP-2 exposure (Fig. 4).

### NMP is osteoinductive *in vivo*

**Fig. 5:**
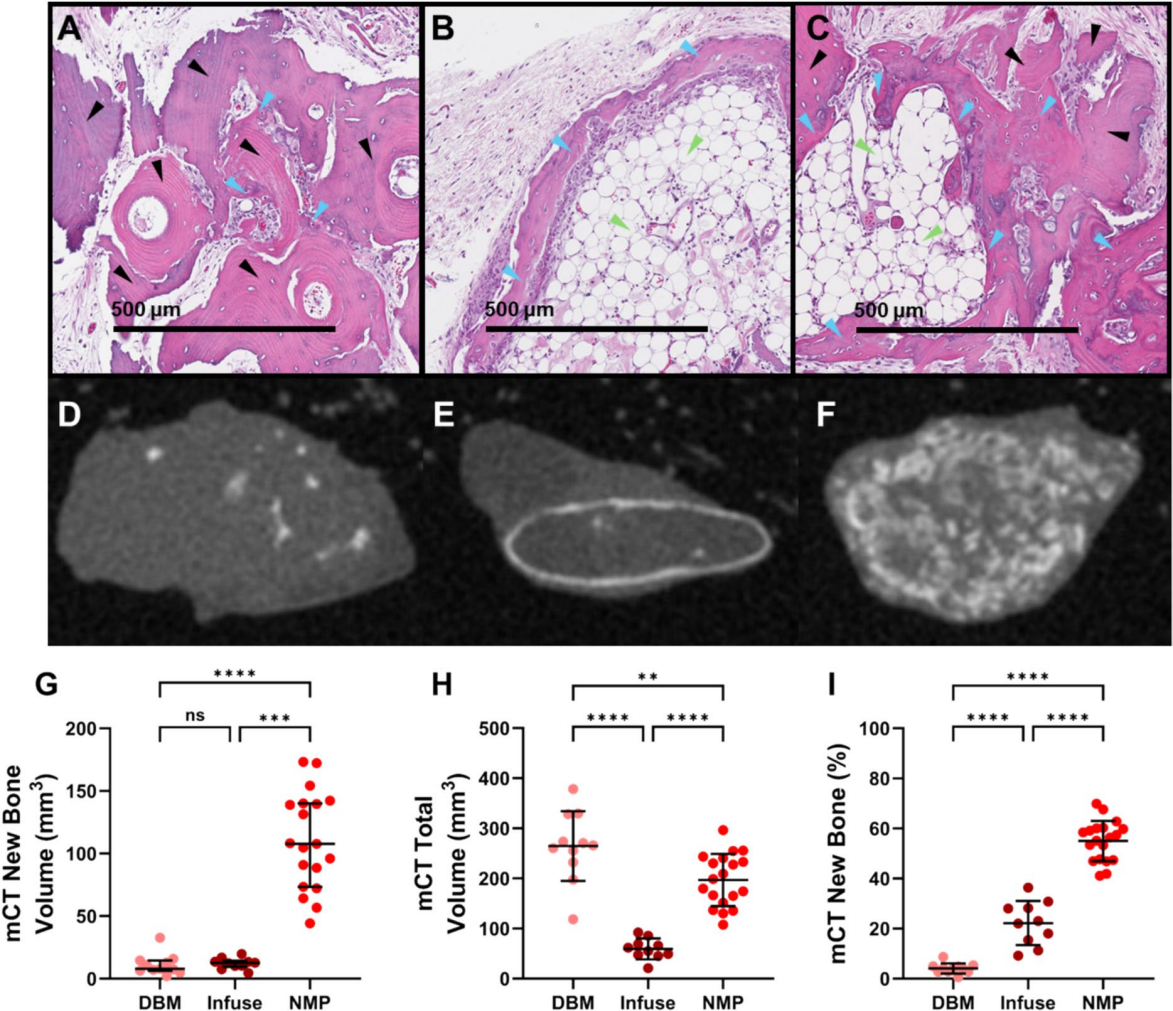
NMP is more osteoinductive than DBM and Infuse *in vivo*. DBM, NMP, or Infuse implants were implanted in rat muscle pouches. Representative histology (A–C; H&E, 40X magnification, and 500 μm scale bar) and microCT reconstructions (D–F) of the recovered 28 day implants are shown for DBM (A, D), Infuse (B, E), and NMP (C, F) explants. Black triangles indicate regions of implanted DBM or NMP, blue triangles indicate regions of new bone, and green triangles indicate regions of bone marrow. Quantification of new bone volume (G), total explant volume (H), and bone volume fraction (I) are presented. Each data point represents a separate implant; data are mean ± SD. Statistical comparisons were made using Kruskal–Wallis with Dunn’s correction (G) or ANOVA with Bonferroni correction (H, I). Adjusted p-values are reported. *p < 0.05, **p < 0.01, ***p < 0.001, ****p < 0.0001, ns: non-significant

As NMP demonstrated osteoinductive potential *in vitro*, we sought to evaluate its osteoinductivity in an rat model, comparing DBM, NMP, and Infuse (rhBMP-2). DBM explants showed non-vital bone matrix surrounded by a mixed cellular infiltrate (Fig. 5A). A few focal areas of new bone and cartilage were present in DBM explants, with an average OI score of 1.18, and only 2 of 11 explants scoring ≥ 2 (Fig. 5A; Table 1). Infuse explants showed a thin ring of new bone surrounding a central core. The core was largely acellular with the appearance of marrow and occasional remnants of ACS with some areas of new bone (Fig. 5B). All Infuse explants were osteoinductive, with a mean OI score of 4 (Table 1). NMP explants showed non-vital bone particles surrounded by large amounts of new bone and with some areas of marrow (Fig. 5C). The average OI score for NMP explants was 3.74, with all explants scoring ≥ 2 and thus being scored osteoinductive (Table 1).

**Table 1:** *In vivo* osteoinductivity histology scoring (ASTM F2529-13)

	Osteoinductivity Score Mean (SD)	Osteoinductive explants (% OI ≥ 2)
DBM	1.18 (0.40) 4.00 (0.00) 3.74 (0.56)	18% (2/11)
Infuse™		100% (10/10)
NMP®		100% (19/19)

In the microCT analysis, the volume of mineralized bone was ~ tenfold higher in NMP explants compared to both DBM and Infuse (*p* < 0.001; Fig. 5G). The total explant volume was highest in DBM, (mean 265 ± 70 mm^3^) (Fig. 5H), followed by NMP (197 ± 52 mm^3^), with Infuse having the smallest volume of recovered implant (59 ± 21 mm^3^). DBM explants were significantly larger than NMP (*p* = 0.004), while the Infuse explants were markedly reduced compared to both DBM and NMP (*p* < 0.001). The new bone fraction was highest in NMP explants (55.03%), followed by Infuse at 22.25%, and lastly DBM at 4.09% (Fig. 5I; all *p* < 0.001).

## Discussion

**Fig. 6:**
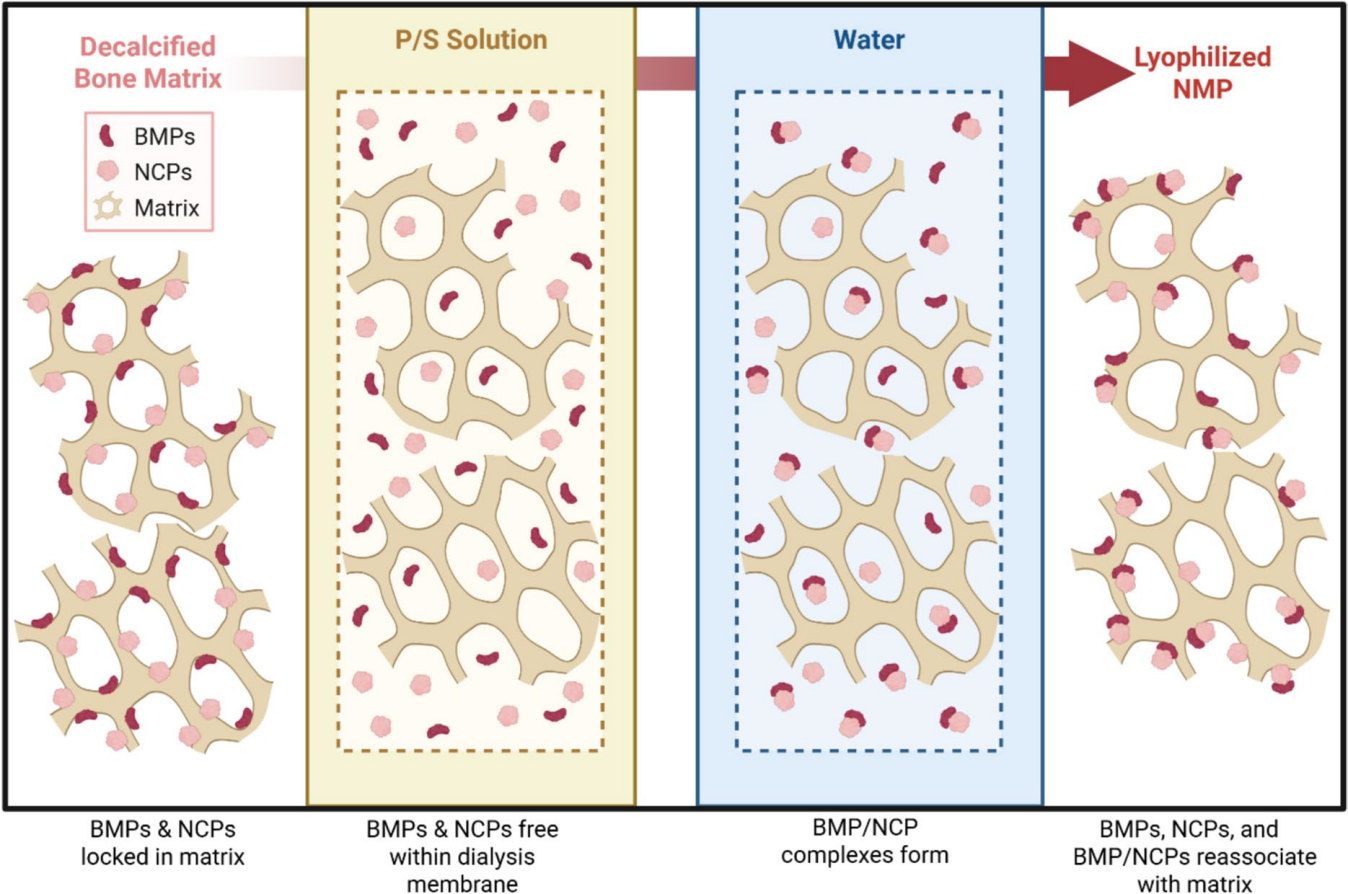
Proposed mechanism of the NMP process. Schematic representation of the hypothesized redistribution and presentation of BMPs and NCPs on the bone particle surface. Panel 1: BMPs and NCPs are entrapped within the decalcified bone matrix. Panel 2: P/S solution treatment within a dialysis membrane (dashed line) releases BMPs and NCPs into the enclosed space. Panel 3: Following water washes, free BMPs and NCPs begin to form complexes. Panel 4: Upon lyophilization, BMP/NCP complexes and remaining free BMPs and NCPs reassociate with the insoluble matrix surface, redistributed into more accessible locations. Created in BioRender. Created in BioRender. Smith, M. (2025) https://BioRender.com/g0q3cyx

The NMP process consistently resulted in an increase in the amount of BMP-7 present in acidic (AcOH) and physiologic (TBS or PBST) extracts compared to DBM from the same donor. In contrast there was no significant difference in the amount of BMP-7 extracted using guanidine. Mechanistically, these findings build upon the two-compartment model of BMP localization in bone matrix (Pietrzak et al. 2012). The original model defined the “tight” compartment as BMPs deeply sequestered within the collagenous matrix, serving as a latent reservoir that becomes available only after repeated chaotrope extraction or significant matrix degradation and a loose compartment from which BMPs were extractable using Gdn and to a lesser extent phosphate buffer. We propose a revised framework with three distinct compartments. First, an inaccessible compartment consisting of BMPs that remain bound within the matrix and are not released, even under strong extraction conditions, but may become available over time, which matches Pietrzak’s tight compartment. Second, we divide Pietrzak’s loose compartment into an extractable compartment representing the total extractable pool of BMPs, as determined by extraction with a strong chaotrope, and a bioavailable compartment. The bioavailable compartment includes proteins more transiently associated with the matrix which are extractable under physiological conditions. Additionally, within the bioavailable compartment, we further distinguish an acid-soluble fraction composed of BMP/non-collagenous protein (NCP) complexes with unique solubility and matrix affinities (Urist et al. 1983) from the BMP soluble at neutral pH. We hypothesize that the NMP process moves BMP from the extractable compartment to the bioavailable compartment and also generates BMP/NCP complexes, thereby expanding the pool of physiologically available growth factors without altering the total BMP content (Fig. 6). This mechanism accounts for the enhanced release of BMP-7 from NMP in physiological buffer extracts (TBS and PBST), while Gdn extracts, which represents the total extractable BMP-7, remain comparable to DBM. The > tenfold enrichment of BMP-7 in the AcOH fraction of NMP provides evidence for the creation of BMP/NCP complexes.

NMP demonstrated significant osteoinductive potential *in vitro*, where incubation of C2C12 cells with NMP resulted in a dose dependant increase in ALP activity (Fig. 4) and incubation of cells with 40 mg/mL of NMP (containing < 10 ng BMP in total) induced a significant increase in ALP activity. C2C12 cells increase ALP in a dose dependent manner when exposed to soluble BMP-2 or -7 across the range of 10 to 5,000 ng/mL (SI Fig. 2), so this elevated activity likely reflects synergistic actions of other factors present in the NMP and prolonged release of BMP from the NMP.

The *in vivo* osteoinductive activity of 0.2 cc of rehydrated NMP (40–50 mg dry) was evaluated in the athymic rat model where it was compared to 105 µg rhBMP-2 on 0.2 cc absorbable collagen sponge (Infuse) and to 0.2 cc of a rehydrated commercial DBM. The athymic rat model is an accepted method for assessing the osteoinductive activity of materials. An ASTM standard has been developed (ASTM Committee F04, 2013) using this model, where the amount of bone forming elements (bone, cartilage or marrow) present in the recovered implant is determined histomorphometrically and requires a minimum score of 2 for the implant to be considered osteoinductive. Based on the ASTM scoring, 100% of the NMP implants tested were osteoinductive, as were the Infuse (rhBMP-2/ACS) implants, while only 18% of the DBM implants were.

We also assessed bone formation in the recovered implants (explant) by microCT, which allowed for assessment of the total volume (TV) of the implant, the volume of mineralized tissue present (BV) through-out the explant, and the bone volume fraction (BV/TV; BVF). The microCT analysis showed NMP produced significantly more mineralized tissue than either DBM or Infuse (Fig. 5G; *p* < 0.001). The volume of the NMP explant (197 mm^3^) was similar to the initial volume implanted (~ 200 mm^3^), the DBM explant volume was significantly larger (265 mm^3^; *p* = 0.004) and the Infuse significantly smaller (59 mm^3^; *p* < 0.001) than NMP; however even when comparing BVF, which normalizes the data to implant volume, NMP formed significantly more bone (55%) compared to Infuse (22%) or DBM (4%) (*p* < 0.001). The discrepancy between histological scoring and microCT analysis (Table 1 and Fig. 5) arises because histological scoring does not consider for differences in volume and measures the presence of bone forming elements (including marrow) rather than bone alone. The Infuse grafts produced an ossicle where an unmineralized core of fatty marrow is surrounded by a thin shell of bone. Histologically, this structure scores as highly osteoinductive, even though the actual amount of bone is minimal. Consistent with this, microCT revealed only a thin shell of calcified bone. Such ossicle-like structures have previously been associated with a large initial release of rhBMP-2 and minimal retention (El Bialy et al. 2017). In contrast, NMP explants exhibited both high histological OI scores and a substantial increase in calcified bone in the microCT analysis, indicating that NMP promotes functional bone regeneration. Differences in explant size (Fig. 5H), as quantified via microCT, may additionally reflect distinct biological processes. The differences in the size of NMP explants as compared to DBM may reflect NMP matrix resorption and bone remodeling, or alternatively may reflect that the DBM underwent further swelling after implantation. Interestingly, the size of Infuse implant was about 86% of the volume of BMP-2 solution added to the ACS, which is what is used as the implant volume estimate clinically.

The 0.2 cc NMP implants contained < 10 ng of BMP-2 and -7 in total, whereas the Infuse implant contained approximately 10,000 times more BMP-2 (105 µg) yet produced less bone. This discrepancy is likely due to several factors. First, NMP releases BMP gradually over at least 12 weeks, with approximately 80% remaining within the matrix after 3 days and 50% remaining at 2 weeks (Fig. 3), a period that coincides with early bone healing when woven bone is initially formed (Kalfas 2001). In contrast, > 95% of BMP is lost from the Infuse implant within 3 days, a time when few, if any, BMP responsive cells are present, and < 1% remains after 2 weeks (El Bialy et al. 2017). Second, the burst release of BMP-2 from the Infuse implant is known to stimulate bone resorption by osteoclasts, which could result in rapid loss of new bone formed, and promote adipogenesis, where mesenchymal stem cells differentiate into adipocytes rather than osteoblasts (James et al. 2016). Finally, NMP contains other growth factors, including TGF-ß1, PDGF, VEGF and IGF-1 and IGF-2 (Kohen et al. 2023) which are known to synergistically stimulate bone formation and therefore may stimulate additional bone formation.

This study also underscores the clinical translational potential of NMP. By enhancing the physiological availability of natural BMPs, NMP does not require the addition of recombinant BMPs to be osteoinductive, thus reducing cost and adverse effects associated with supratherapeutic BMP administration (Carragee et al. 2011). Moreover, the preservation of native protein complexes suggests that NMP could more closely mimic natural bone healing processes, making it an attractive candidate for clinical applications (Bessho 1996).

Bone graft substitutes are typically used in patient populations who are older and have a high incidence of comorbidities and lifestyle factors that impair bone healing such as diabetes, chronic inflammatory conditions, being obese, and being a current smoker (Claes et al. 2012; Copuroglu et al. 2013; Ding et al. 2020; Mahajan et al. 2021; Pollock et al. 2020). A recent study evaluated the safety and effectiveness of NMP clinically in 50 patients undergoing lumbar fusion. These subjects had a mean age of 61.5yrs and a high (98%) rate of comorbidities including diabetes, hypertension, arthritis, and smoking. High fusion rates were demonstrated, similar to those reported for iliac crest bone graft (Nunley et al. 2025), suggesting that NMP is effective in such populations. Additional clinical studies are ongoing which will allow for further evaluation of the impact of various comorbidities on the safety and effectiveness of NMP.

Strengths of this study include reproducibility across multiple bovine lots, consistent findings that translate from *in vitro* to *in vivo*, and the combination of histological and quantitative microCT analyses. Limitations include the small number of human donors evaluated, the need for longer-term *in vivo* studies to assess remodeling, and the absence of direct evidence supporting the BMP/NCP distribution hypothesis. Future studies should systematically evaluate donor variability, determine clinically relevant osteoinductive cutoffs (Murray et al. 2007), use immunostaining to assess BMP distribution, and explore NMP performance in clinical models.

In summary, the NMP process enhances BMP bioavailability and formation of BMP/NCP complexes associated with the matrix, resulting in robust and sustained osteoinductive activity. By improving the accessibility and presentation of growth factors, NMP promotes functional bone formation that is superior to conventional DBM and comparable or superior to recombinant BMP. These findings support further investigation of NMP as a clinically relevant, next-generation bone graft material. The results reported here support the hypothesis that redistributing BMP to the bioavailable compartment increases its physiologic release, thereby resulting in a more osteoinductive allograft.

## Conclusions

The NMP process produces BMP/NCP complexes and enhances the bioavailability of the natural BMPs, resulting in a matrix with robust and sustained osteoinductive activity. NMP has significantly increased BMP-7 bioavailability compared to donor-matched DBM controls, stimulates dose-dependent ALP activity in C2C12 cells, and produces more mineralized bone *in vivo* than either a commercial DBM or recombinant BMP-2 (Infuse). By improving the accessibility and presentation of growth factors, NMP promotes functional bone formation and represents a next-generation, clinically relevant bone graft material.

## Supplementary Information

Below is the link to the electronic supplementary material.

Supplementary file1 (DOCX 105 KB)

## Data Availability

All data supporting the findings of this study will be made available upon reasonable request to the corresponding author (Sean Peel; sean.peel@redrockregen.com). All primary data necessary to replicate the results and figures presented in this manuscript are retained by the authors. The provided data are intended solely for non-commercial, academic research purposes. Data access will be granted under a standard Data Transfer Agreement (DTA) to ensure appropriate acknowledgement and adherence to ethical and institutional guidelines.
