# Supplementary material for: Development of a growth factor bioavailability enhanced allograft (GFBA) for bone regeneration
Source: Cell Tissue Bank. 2026 Jan 22;27(1):6. doi: 10.1007/s10561-025-10206-y (PMC12827305; doi:10.1007/s10561-025-10206-y)

Development of a Growth Factor Bioavailability Enhanced Allograft (GFBA) for Bone Regeneration

Authors:

Marie-Soleil R. Smith^1^, Sowmya Shivanna^1^, Yakup Kohen^1,2,3^, Shiva Naseri^1^, Yasmin Mawani^1^, Sean A.F. Peel^1,4^

Affiliations

1 Red Rock Regeneration Inc, Toronto, ON, Canada

2 Department of Medical Biophysics, Temerty Faculty of Medicine, University of Toronto, Toronto, ON, Canada

3 Princess Margaret Cancer Centre, University Health Network, Toronto, ON, Canada

4 Faculty of Dentistry, University of Toronto, Toronto, ON, Canada

Corresponding Author:

[sean.peel@redrockregen.com](mailto:sean.peel@redrockregen.com)

**Journal:**

Cell and Tissue Banking

**Supplementary Information**

Fig. 1 Correlation of BMP-7 content in different extract solutions


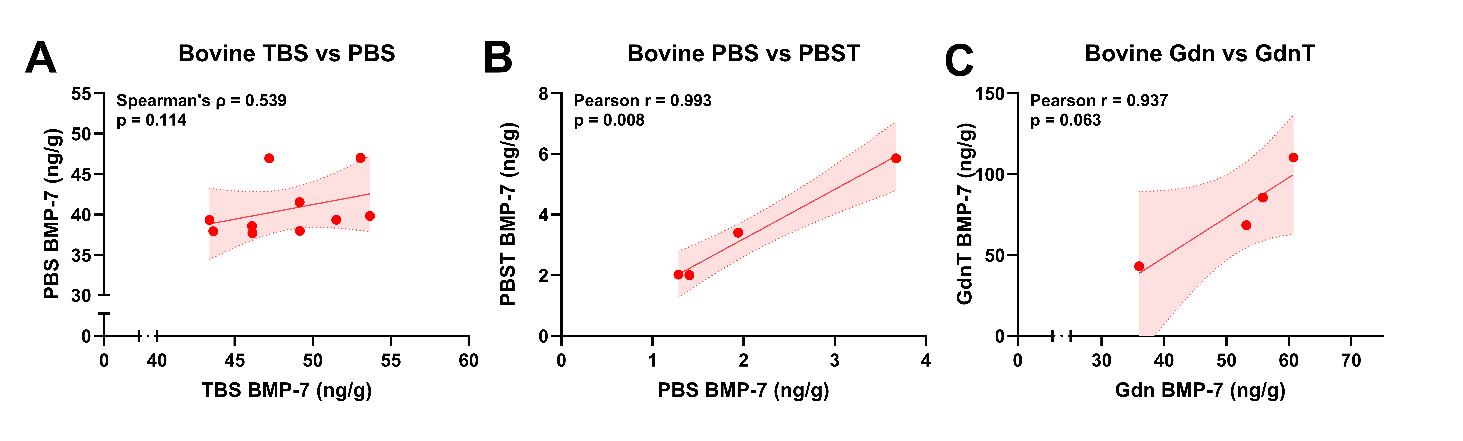


Fig. 2 Dose-dependent ALP increase in C2C12 cells with BMP-2 or BMP-7 treatment


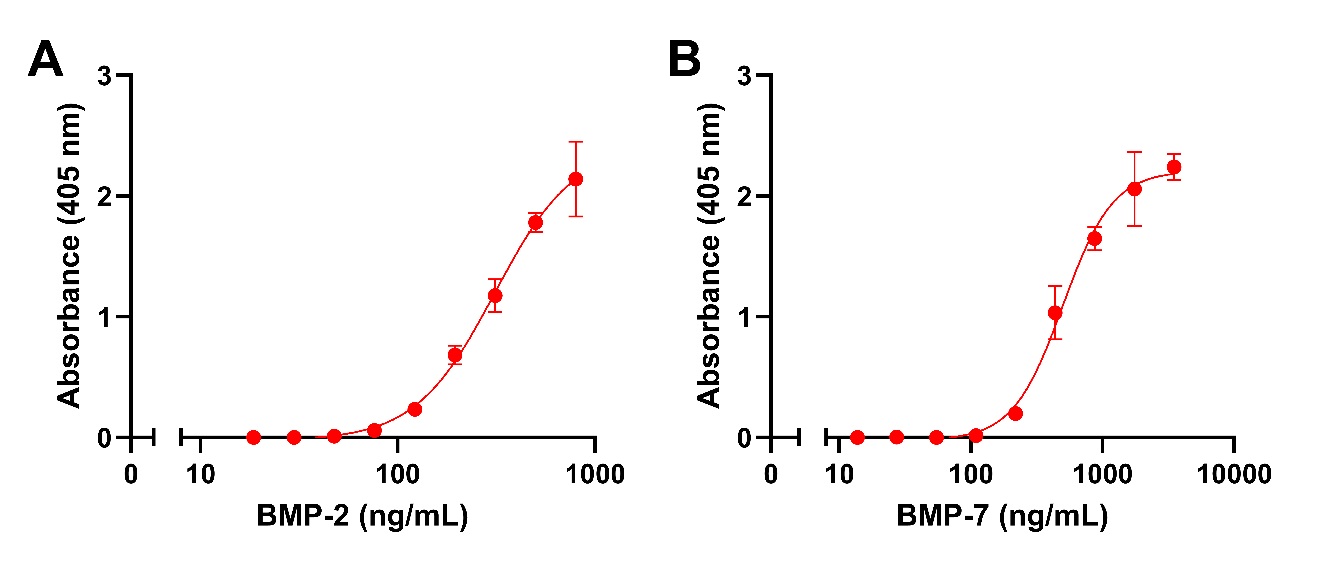

Supplement: Supplementary file 1 — Supplementary file1 (DOCX 105 KB) [file 10561_2025_10206_MOESM1_ESM.docx]
